# MRI-guided transurethral ultrasound prostate ablation: midterm outcomes of a phase I clinical trial

**DOI:** 10.1186/2050-5736-3-S1-O60

**Published:** 2015-06-30

**Authors:** Mathieu Burtnyk, Michele Billia, Ionel Valentin Popeneciu, Jason Hafron, Matthias Roethke, Heinz-Peter Schlemmer, James Relle, Sascha Pahernik, Joseph Chin

**Affiliations:** 1 Profound Medical Inc., Toronto, Canada; 2 London Health Sciences Center, London, United Kingdom; 3 Heidelberg University Hospital, Heidelberg, Germany; 4 Beaumont Health System, Royal Oak, Michigan, United States; 5 German Cancer Research Center, Heidelberg, Germany

## Background/introduction

MRI-guided transurethral ultrasound ablation (TULSA) is a new minimally-invasive modality for the treatment of prostate cancer, which aims to provide local disease control with low morbidity. A transurethral ultrasound device generates a continuous volume of thermal coagulation that is shaped precisely to the prostate using real-time MR thermometry and active temperature feedback control. The aim of this multi-center, prospective Phase I clinical study is to determine the safety and feasibility of MRI-guided TULSA, and to assess initial efficacy for treatment of localized prostate cancer.

## Methods

A total of 30 patients were enrolled with biopsy-proven, low-risk, localized prostate cancer: age ≥ 65 years, clinical stage T1c/T2a, PSA ≤ 10ng/ml, Gleason Score ≤ 3+3 (3+4 max in Canada only). Treatment was completed under general anesthesia and drainage from a suprapubic catheter (SPC) which remains for 2 weeks. Treatment planning was performed under MRI prostate visualization, with therapeutic intent of whole–gland ablation. Treatment was delivered under continuous MR thermometry active feedback control. Primary endpoints are safety and feasibility, with follow-up to 12 months. Complete clinical monitoring is 5 years, including serial PSA, completion of quality-of-life-questionnaires and prostate biopsy at 12 months.

## Results and conclusions

Median (range) prostate volume and treatment time were 47 (21-95) cc and 36 (24-61) min, respectively (n=30). MR thermometry measurements depict a continuous region of heating with a high degree of spatial control of the ablation volume, to within 0.1 ± 1.3 mm (n=30). Median PSA reduced by 90% (60 – 99%) to 0.7 ng/ml at 1 month (n=28), remaining stable to 0.6 ng/ml at 6 months (n=20). MRI-guided TULSA was well-tolerated by all patients, with no intraoperative complications, and no reported cases of urinary incontinence, fistula or rectal injury. All complications to-date were CTCAE v4 Grade 1-3 and included: hematuria (15), urinary tract infection (10), epididymitis (1), and acute urinary retention (4) requiring prolonged or re-catheterization. Normal micturition returned after SPC removal, with return to baseline by 3 months (n=26) and improvement by 6 months (n=21): IPSS median score 9 (baseline) to 6 (6 months), and peak urinary flow 14 ml/s (baseline) to 19 ml/s (6 months). MRI-guidance enables accurate planning and real-time dosimetry and control of the thermal ablation volume. Midterm results indicate that MRI-guided TULSA is safe and clinically feasible with a well-tolerated, low side effect profile.

